# The impact of disruptions due to COVID‐19 on HIV transmission and control among men who have sex with men in China

**DOI:** 10.1002/jia2.25697

**Published:** 2021-04-06

**Authors:** Ross D Booton, Gengfeng Fu, Louis MacGregor, Jianjun Li, Jason J Ong, Joseph D Tucker, Katherine ME Turner, Weiming Tang, Peter Vickerman, Kate M Mitchell

**Affiliations:** ^1^ University of Bristol Bristol United Kingdom; ^2^ MRC Centre for Global Infectious Disease Analysis Imperial College London London United Kingdom; ^3^ Jiangsu Provincial Center for Disease Control and Prevention Nanjing China; ^4^ Social Entrepreneurship to Spur Health (SESH) Global Guangzhou China; ^5^ Faculty of Infectious and Tropical Diseases London School of Hygiene & Tropical Medicine London United Kingdom; ^6^ Central Clinical School Monash University Melbourne Australia; ^7^ University of North Carolina Project‐China Guangzhou China; ^8^ University of North Carolina at Chapel Hill Chapel Hill NC USA

**Keywords:** COVID‐19 pandemic, men who have sex with men, modelling, HIV transmission, key and vulnerable populations, People’s Republic of China

## Abstract

**Introduction:**

The COVID‐19 pandemic is impacting HIV care globally, with gaps in HIV treatment expected to increase HIV transmission and HIV‐related mortality. We estimated how COVID‐19‐related disruptions could impact HIV transmission and mortality among men who have sex with men (MSM) in four cities in China, over a one‐ and five‐year time horizon.

**Methods:**

Regional data from China indicated that the number of MSM undergoing facility‐based HIV testing reduced by 59% during the COVID‐19 pandemic, alongside reductions in ART initiation (34%), numbers of all sexual partners (62%) and consistency of condom use (25%), but initial data indicated no change in viral suppression. A mathematical model of HIV transmission/treatment among MSM was used to estimate the impact of disruptions on HIV infections/HIV‐related deaths. Disruption scenarios were assessed for their individual and combined impact over one and five years for 3/4/6‐month disruption periods, starting from 1 January 2020.

**Results:**

Our model predicted new HIV infections and HIV‐related deaths would be increased most by disruptions to viral suppression, with 25% reductions (25% virally suppressed MSM stop taking ART) for a three‐month period increasing HIV infections by 5% to 14% over one year and deaths by 7% to 12%. Observed reductions in condom use increased HIV infections by 5% to 14% but had minimal impact (<1%) on deaths. Smaller impacts on infections and deaths (<3%) were seen for disruptions to facility HIV testing and ART initiation, but reduced partner numbers resulted in 11% to 23% fewer infections and 0.4% to 1.0% fewer deaths. Longer disruption periods (4/6 months) amplified the impact of disruption scenarios. When realistic disruptions were modelled simultaneously, an overall decrease in new HIV infections occurred over one year (3% to 17%), but not for five years (1% increase to 4% decrease), whereas deaths mostly increased over one year (1% to 2%) and five years (1.2 increase to 0.3 decrease).

**Conclusions:**

The overall impact of COVID‐19 on new HIV infections and HIV‐related deaths is dependent on the nature, scale and length of the various disruptions. Resources should be directed to ensuring levels of viral suppression and condom use are maintained to mitigate any adverse effects of COVID‐19‐related disruption on HIV transmission and control among MSM in China.

## 1 INTRODUCTION

Globally, 37.9 million people are living with HIV (PLHIV) [1], with men who have sex with men (MSM) disproportionally affected [2]. In China, a recent systematic review indicated an HIV prevalence of 5.7% (95% CI: 5.4% to 6.1%) among MSM, with increasing HIV prevalence in this group over time (2001 to 2018) [3], whereas HIV incidence in this population has fluctuated over time (Beijing, 2008 to 2016) [4]. Efforts to manage the HIV epidemic in China have been made increasingly difficult by the COVID‐19 pandemic [5, 6], having significant potential to affect the HIV care continuum and patterns of sexual risk behaviour in numerous settings worldwide [7, 8, 9, 10, 11]. Close examination of this syndemic is a key issue for global public health [12].

Among PLHIV in China, who already face high levels of HIV stigma, psychological distress and suboptimal adherence to antiretroviral therapy (ART) [5], the COVID‐19 pandemic has presented further barriers to HIV prevention and treatment [6]. Quarantine and physical distancing and reduced testing capacity (as staff are diverted to COVID‐19 responses) reduces the identification and treatment of new HIV infections [13]. Timely linkage to HIV services and initiation of ART have been affected during the COVID‐19 pandemic, with many hospitals designated for treatment of COVID‐19 suspending taking on new patients with HIV [5]. The COVID‐19 pandemic has hindered ART, due to hospital visits being restricted from city lockdowns/traffic controls [5]. In February 2020, a survey in China found 32.6% of PLHIV were at risk of ART discontinuation and about half (48.6%) did not know where to get ART in the near future [14]. These gaps in HIV treatment could lead to increased HIV‐related deaths and a higher risk of HIV transmission. China reported disruptions in HIV provision between April and June 2020 [15]. MSM in other countries have reported having fewer sexual partners during periods of COVID‐19‐related lockdown, which may temporarily reduce HIV transmission [8].

Mathematical modelling can be used to capture the complexity of these changes and estimate the impacts of COVID‐19 on HIV epidemiology. One modelling study of PLHIV in Africa projected that a six‐month interruption in ART supply across 50% of the population on treatment could lead to a 60% increase in HIV‐related deaths over a one‐year period [16]. Another modelling study on low‐and‐middle‐income countries (LMIC) projected that HIV‐related deaths would increase by 10% over the next five years, with the greatest impact on mortality estimated to be from ART interruptions [17]. However, neither of these studies used quantitative COVID‐19 impact data to inform their modelled disruptions, which is essential for obtaining reliable projections for the true scale of COVID‐19 disruptions.

To our knowledge, no COVID‐19 impact modelling has been published for LMIC focussing on key populations, who are the main groups affected by HIV [18]. In addition, no model projections have to date incorporated observed quantitative data from the COVID‐19 disruption. In this study, we addressed this by collating data on the impact of COVID‐19 and resulting lockdown measures on ART initiation and viral suppression among PLHIV and on HIV testing, sexual risk behaviour and condom use among MSM in China. We used a model of HIV transmission/treatment among MSM in China to estimate the impact of these disruptions on new HIV infections and HIV‐related deaths. We identified which aspects of HIV prevention and treatment should be prioritized to mitigate the adverse effects of COVID‐19.

## 2 METHODS

### 2.1 Observed disruptions due to COVID‐19 in China

Estimates of HIV testing (among MSM), treatment initiation and viral load suppression (among PLHIV) came from surveillance data from Jiangsu province (HIV testing/clinics), from the first quarter of 2019 and 2020 [19]. Estimates of changes in a number of all sexual partners and consistency of condom use came from a different, independent online survey conducted among MSM (N = 731) across 31 provinces in China between 18 May and 2 June 2020, during the COVID‐19 pandemic. From these data, we estimated the following percentage changes due to the disruption caused by COVID‐19, compared to the pre‐COVID period (before 1 January 2020):

- The number of MSM undergoing facility‐based HIV testing in the first quarter of 2019 was 6436 compared to 2641 in 2020, a reduction of 59% (3795/6436; 95% CI:58% to 60%).
- The number of PLHIV initiating ART in the first quarter of 2019 was 315 compared to 208 in 2020, a reduction of 34% (107/315; 95% CI:29% to 39%).
- There was no change in viral suppression (VS) among PLHIV. 95.3% (940/986; 95% CI:93.8% to 96.6%) of viral load tests showed VS in the first quarter of 2019, with similar numbers in 2020 (96.0%, 928/967; 95% CI:94.5% to 97.1%). The proportion of diagnosed PLHIV who had a viral load test was similar in both years: 4.7% in the first quarter of 2019 and 4.3% in the first quarter of 2020. Note most viral load tests in this region are conducted in the third/fourth quarter.
- 62% of MSM (313 of 506 MSM who had male partners in the last six months) reported reduced numbers of sexual partners (in the last three months) compared to the pre‐pandemic period.
- 25% of MSM (126 of 506 MSM who had male partners in the last six months) used condoms less with their partners (in the last three months, all partnerships) compared to the pre‐pandemic period.

### 2.2 Mathematical model

We used a model of HIV testing/transmission/treatment among MSM in China which was developed to evaluate the long‐term impact of an HIV‐testing intervention in eight cities in China [20]. The four cities we study (Guangzhou/Shenzhen/Jinan/Qingdao) were the largest cities in the randomized control trial, had the highest quality data available and represent two regions (Guangdong and Shandong). There was no modelled migration between the four cities. All individuals are categorized by infection status, risk (≤/>two male anal sex partners, last three months), anal sex role (always insertive/versatile/always receptive), infection stage (acute/CD4 >500/351‐500/200‐350/<200 cells/µL) and diagnosed/ART status. Those not on ART move into more advanced stages of infection, whereas those on ART do not; their mortality is modelled as a function of infection stage at ART initiation (Figure S2).

Facility‐based and self‐testing are modelled. MSM are distinguished by whether or not they have previously tested. HIV transmission occurs via anal sex between MSM which depends on HIV disease stage/ART coverage/VS/total partners/total sex acts/sexual role/condom efficacy and use. The model was calibrated to city‐level HIV epidemics, using demographic/behaviour data from CDC/trials, calibrated to local city/province/national‐level estimates (“fitting metrics”) of HIV prevalence/ART coverage/diagnosis/incidence/population size [20]. Model/schematics (Figures S2,S3), parameters/fitting metrics (Table S6), time‐dependent testing assumptions (Figure S6) and model projections (Figure S1) are given in the supplement.

### 2.3 Base case scenario (no COVID‐19)

The model was run for five years until 1 January 2025 using the fitted model parameters, with all parameters constant at their 2019 values from 2020 onwards. These base case runs predicted the non‐COVID‐19 trajectory for each city.

### 2.4 COVID‐19‐related disruption scenarios

Disruption scenarios were implemented from 1 January 2020 and run for 3/4/6‐month disruptions, after which all parameters were reset to their original pre‐COVID‐19 values. Comparisons of these scenarios with the base case were made over one (1 January 2021) and five years (1 January 2025).

The following *observed* disruption scenarios were based on the observed disruptions – reductions in:

- facility‐based HIV testing (59%)
- ART initiation (34%)
- number of sexual partnerships (31% to 62% (assuming 50% to 100% reductions among those with fewer partners)).
- condom use (12.5% to 25% (assuming 50% to 100% reductions among those with reduced use)).

Although data from Jiangsu province suggested no disruption to VS, disruptions in ART provision have been reported to the WHO [15]. We explored an additional *hypothetical* scenario where VS was reduced by 10% (consistent with reductions in ART access among MSM in the United States [8]) and 25% (consistent with disruptions to ART uptake reported among PLHIV in China [5]):

- Reduction in VS of 10/25%

The data on sexual partnerships/condom use estimated the proportion of MSM having fewer partnerships/using condoms less frequently (not overall reductions in numbers of partners/condom use). We assumed those reporting fewer partnerships/less condom use reduced their partnerships/condom use by 50% to 100% to account for uncertainty in reductions to partner numbers/condom use. Reductions in condom use were modelled as a reduction in the proportion of sex acts in which a condom is used. Reductions in HIV testing/ART initiations were modelled as reductions in facility‐based HIV testing/ART initiation rates, and reductions in partner numbers were modelled as reductions in numbers of partners per year (across risk groups). VS reductions were modelled as increases in infectiousness and HIV‐related mortality among those on ART, assuming a proportion (10/25%) of virally suppressed MSM stop taking ARV, having the same infectiousness/HIV‐related‐mortality as individuals, not on ART. No reduction in HIV self‐testing rates was modelled, in line with observations in Jiangsu [21].

We assessed the impact of each disruption separately (A, B, C, D, E), and the combined impact occurring simultaneously (with/without scenario‐E). The impacts of disruptions lasting 3/4/6 months were assessed (after which parameters return to pre‐COVID disruption levels).

Outcome measures used were total and relative percentage change in new infections and HIV‐related deaths, compared to the base‐case non‐COVID‐19 scenario, evaluated over one and five years from 1 January 2020. Impact measures were expressed as median values and 95% credible intervals (95% CrI), across 100 selected parameter sets in each city (which maximize the log‐likelihood from 100,000 Latin Hypercube samples [20]), and across 400 parameter sets from all cities.

We analysed the sensitivity of these scenarios (A, B, C, D, E) to different magnitudes of disruption (0, 25, 50, 75, 100%) over one and five years with a three‐month disruption (example disruption period). We plot the percentage change in new HIV infections and HIV‐related deaths as a function of each individual disruption parameter.

## 3 RESULTS

The percentage change in new HIV infections and HIV‐related deaths did not vary between each city, with greater within‐city variation across the scenarios. Therefore, all results are presented as the overall impact across four cities (Table S1), with separate cities in Tables S2‐S5.

### 3.1 Single and combined three‐month disruptions

**Figure 1:**
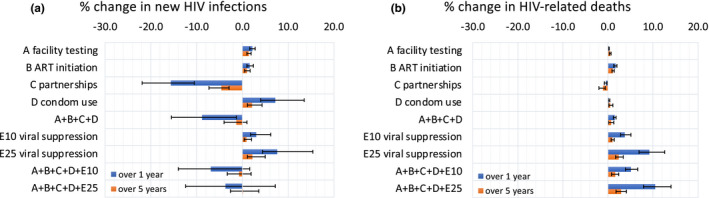
The percentage change in **(a)** new HIV infections and **(b)** HIV‐related deaths under disruption scenarios evaluated over a one‐ and five‐year time horizon (blue and orange respectively) in four cities in China. Bars indicate median values, while error bars show the 95% credible intervals for each scenario and time horizon. Scenarios are as follows: (A) Reduction in facility‐based HIV testing (59%), (B) Reduction in ART initiation (34%), (C) Reduction in number of sexual partnerships (31% to 62%), (D) reduction in condom use (12.5% to 25%) E10) Reduction in viral suppression of 10%, E25) Reduction in viral suppression of 25%.

Realistic disruptions to facility‐based HIV testing, ART initiation and condom use increased new HIV infections among MSM (Figure 1a, Tables S1‐S5). Disruptions to condom use (scenario‐D) lasting three months led to the largest relative increase in HIV infections, of 7.8% (95% CrI:4.5% to 13.8%) over one year (Figure 1a), with relative increases of 2.3% (1.7% to 2.9%) and 1.7% (1.2% to 2.4%) predicted over one year for realistic three‐month disruptions to facility‐based HIV testing (scenario‐A) and ART initiations (scenario‐B) respectively. Reductions in numbers of sexual partners (scenario‐C) reduced HIV infections by 16.2% (11.1% to 23.2%) over one year following a three‐month disruption.

Hypothetical 10/25% reductions in VS (E10/E25) increased numbers of HIV infections. 25% VS reductions increased new HIV infections by 7.4% (4.7% to 14.0%) over one year given a three‐month disruption.

The effect of disruption scenarios on the relative percentage change in HIV infections was always smaller over five years than one year (but not the absolute difference in HIV infections, which generally increased over five years), with a more rapid decrease in effect over five years seen for disruptions to partnership numbers, condom use and VS (Figure 1a).

For simultaneous disruptions A + B + C + D, new HIV infections decreased over one year (median 8.7%, (2.8% to 17.2%)), but over five years reduced to 1.6% (−0.6% to 4.3%) due to disruptions to HIV testing (increase 1.7%, 1.2% to 2.4%) and ART initiation (increase 1.1%, 0.7% to 1.8%) having longer‐lasting effects on ART outcomes, with ART taking longer post‐disruption to return to pre‐disruption levels.

HIV‐related deaths increased following disruptions to HIV testing/ART initiations/condom use/VS (scenarios‐A, B, D, E), and decreased following disruptions to partnerships (scenario‐C) (Figure 1b, Tables S1‐S5). Small impacts (<1%) on HIV‐related deaths were predicted over one year for three‐month disruptions to HIV testing/partner numbers/condom use. Larger increases in HIV‐related deaths were predicted to occur following three‐month disruptions to ART initiations – a 1.8% (1.5% to 2.0%) increase over one year – and, especially, VS – a 10.1% (7.6% to 12.7%) increase over one year following 25% VS reductions. The observed disruptions (A + B + C + D) resulted in 1.5% (1.1% to 1.8%) more HIV‐related deaths over one year and 0.6% (−0.3% to 1.2%) over five years.

Including reductions of 10/25% in levels of VS alongside the other, observed scenarios (A + B + C + D + E10, A + B + C + D + E25) always led to an increase in new HIV infections, and, particularly, an increase in HIV‐related deaths compared to the observed scenarios alone (A + B + C + D) (Figures 1a,b).

### 3.2 Sensitivity in disruption duration

**Figure 2:**
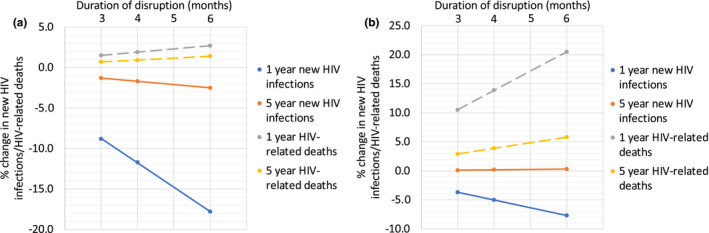
The percentage change in new HIV infections and HIV‐related deaths for scenarios **(a)** A + B + C + D and **(b)** A + B + C + D + E25 for varying disruption periods (3, 4 and 6 months) and time horizons (one‐ and five‐year) in four cities in China. Dots indicate median values.

When comparing the impacts of two combined disruption scenarios (A + B + C + D and A + B + C + D + E25) for disruptions lasting 3/4/6 months, we found impacts on new HIV infections and HIV‐related deaths were approximately linear, with a four‐month disruption leading to around 34% greater impact than a three‐month disruption, and a six‐month disruption around two times the impact of a three‐month disruption, over both one‐ and five‐year time horizons (Figure 2).

### 3.3 Impact in different cities

**Figure 3:**
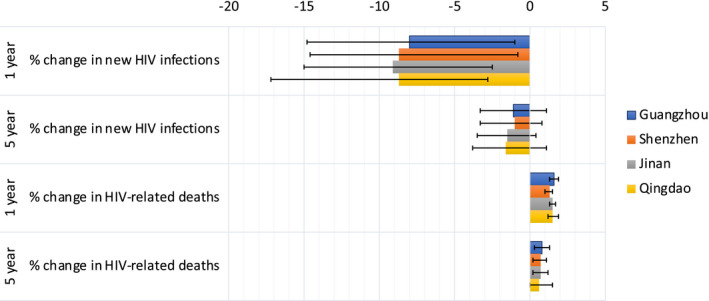
The percentage change in new HIV infections and HIV‐related deaths for scenario A + B + C + D for different cities (Guangzhou, Shenzhen, Jinan and Qingdao), and time horizons (one‐ and five‐year). Bars indicate median values and error bars show the 95% credible intervals.

Absolute numbers of predicted additional/prevented infections and deaths varied between cities (Tables S2‐S5), related to differing MSM population sizes. Percentage changes in infections and deaths did not vary substantially between cities (Figure 3, Tables S2‐S5). For example, for A + B + C + D, the reduction in new HIV infections over one year varied from 8.7% (−2.8% to 17.2%) in Qingdao to 9.1% (2.5% to 15.0%) in Jinan, with far greater within‐city than between‐city uncertainty (Figure 3).

Over five years, realistic three‐month disruptions to HIV testing/ART initiations/sexual risk behaviour/condom use (scenario‐A + B + C + D) would lead to on average three fewer new HIV infections but 1 additional HIV‐related death among MSM in Jinan and Qingdao, six fewer infections and three additional deaths in Guangzhou, and to 18 fewer HIV infections but nine additional HIV‐related deaths among MSM in Shenzhen.

The combined scenario (A + B + C + D + E) for three months over five years, would lead to an average three additional new HIV infections in Guangzhou, seven in Shenzhen but no change in Jinan and Qingdao, with 11 additional HIV‐related deaths in Guangzhou, 33 in Shenzhen and three in Jinan and Qingdao.

### 3.4 Sensitivity in disruption magnitude

Over one or five years, with a three‐month disruption, the relationship between the magnitude of the disruption (0%, 25%, 50%, 75%, 100%) and the projected impact was always linear (Figure S4,S5; Table S7,S8). Greater disruption led to increases in impact measures for four disruption parameters (facility testing/ART initiation/condom use/VS) but not for partnerships (fewer infections and deaths).

For a three‐month disruption evaluated over a one‐year, theoretical disruption scenarios A50‐E50 (affecting 50% of MSM) increased new HIV‐infections by 1.8%, 2.4%, −16.7% (decrease), 15.8% and 11.3%, respectively, and increased HIV‐related deaths by 0.1%, 2.5%, −0.4% (decrease), 0.5% and 18.2% respectively (Figure S4; Table S7). If disruption scenarios affected 100% of MSM (A100‐E100), new HIV‐infections were projected to increase to 3.7%, 4.9%, −30.2% (decrease for 95% affecting MSM), 38.7% and 29.3% respectively. Scenarios A100‐E100 increased HIV‐related deaths to 0.3%, 5.3%, −0.8% (decrease – for 95% disruption), 1% and 35.7% respectively.

## 4 Discussion

Available data in this analysis suggest the COVID‐19 pandemic and measure undertaken against it have resulted in reduced rates of HIV testing and treatment among MSM in this region of China but have not had an impact on VS rates (as of mid‐2020). Survey data suggested MSM in China had fewer partners and used condoms less often during the COVID‐19 pandemic. Using these data, simulating realistic three‐month disruptions to HIV testing/ART initiation/condom use/partner numbers, fewer new HIV infections are projected to occur among MSM in China over 2020 (9% fewer) than would have occurred in the absence of the COVID‐19 pandemic, with a smaller decrease (2%) seen over five years. This decrease was largely due to reductions in sexual partner numbers counteracting a reduction in ART initiations and condom use. Our model suggests these disruptions will lead to small increases in HIV‐related deaths (2% over one year, 1% over five years). New HIV infections were most adversely affected by disruptions in condom use and VS, and HIV‐related deaths by reductions in VS. Therefore, our results suggest HIV prevention and treatment efforts should focus on maintaining the use of condoms and VS among MSM in China to mitigate short/long‐term effects of the COVID‐19 disruption.

The length of disruption is also critical in determining the longer‐term impacts of COVID‐19. Sensitivity analyses (with longer four/six‐month disruptions) demonstrated a linear relationship between disruption duration and impact. The direction of the relationship was different for HIV‐related deaths (positive – longer duration gave more deaths) and new HIV infections (negative – longer duration gave fewer new infections).

The total new HIV infections and HIV‐related deaths varied between the four cities in China, due to different population sizes, epidemiology and care cascades in each city (reflected in calibration data [20]). However, the percentage change in impact measures did not vary between each city, with much greater within‐city variation. This result is surprising, considering each city has a different future projected HIV prevalence (5.0% to 12.2% in 2036, based on data specific to these cities [22] in Booton et al. [20]) and are in two separate provinces (Guangdong/Shandong). Therefore, the impact predicted in this study is likely to be applicable to MSM within any region in China.

We may compare our results to other modelling studies of COVID‐19‐related disruption on HIV prevention and treatment. Jewell et al. [16] used multiple African models of three‐month disruptions affecting 50% from 1 April 2020, reporting increases in HIV incidence of <1% from the suspension of HIV testing (China model; 1% to 2% for 50% reductions in facility testing, scenario‐A50, Table S7), <2% from no new ART initiations (2% to 4%, scenario‐B50), 2% to 9% from the interruption of condom availability (12% to 33%, scenario‐D50), 4% to 89% from ART interruption (9% to 31% for 50% reduction in VS, scenario‐E50). Suspension of testing for 50% increased deaths by <1% (China model; <1%, scenario‐A50), ART initiation <2% (2% to 3%, scenario‐B50) and condom availability 0% (0% to 1%, scenario‐D50) with ART interruption causing an increase of 17% to 62% (14% to 25%, scenario‐E50). Our results align well with these estimates, considering the different methodology/definitions of disruption/population (all adults/children, compared to solely MSM) and underlying models and data (different settings/treatment/condom use). This is a major strength of our study – the use of early data from China to estimate data‐driven (rather than theoretical) magnitudes of COVID‐19‐related disruptions. Another strength of this study was performing our analysis on four separate cities from two distinct regions within China. In addition, our analysis involves various scenarios and the effects of combining these enables us to better understand the relative impact of different disruptions.

Our analysis has some limitations which should be acknowledged. Not all of the disruption estimates were MSM specific, and MSM may have had increased disruption when compared to other populations (MSM facility testing was reduced by 59%, compared to 29% for the entire population [19]). We may have underestimated the disruption to ART initiations. The survey data only gave semi‐quantitative estimates of disruptions to partnerships and condom use that is the proportion of MSM using fewer condoms with their partners/having fewer partners and not the overall reduction in condom use/partner numbers. To address this issue, we explore uncertainty in the reduction of condom use and partnerships by sampling from a wider distribution of estimates. A disruption in the number of partnerships could act as a temporary delay in initiating partnerships, but with our data and model, we are unable to assess this. If partnerships are delayed rather than forgone, smaller impacts on new HIV infections would be expected. Future data on partnerships during and after the COVID‐19 pandemic would enable models to more accurately capture changes in MSM behaviour. Furthermore, the disruption estimates for testing/ART initiations/VS came from Jiangsu, different to the cities we model (Guangdong/Shandong), and estimates for disruptions to partner numbers/condom use came from 31 provinces, meaning we may not have fully captured the impact of COVID‐19 in each city. In addition, the data were only collected at a single point in time, so we cannot account for changes over the course of the epidemic. We have not modelled the direct impacts of COVID‐19 infection, or the effects of pre‐exposure prophylaxis (PrEP), which has only recently been licensed in China or of those MSM who may not be registered as residents in the district/province in which they are currently living which would reduce the individuals’ access to ART (as provision is regulated such that individuals must be registered as residents).

Future extensions to this work could include modelling, the potential characteristics of co‐infection between HIV and COVID‐19, and the effects of PrEP. Future work should address longer‐term impacts of COVID‐19 on HIV incidence and mortality over 10/20 years, calculate the resources needed to offset any adverse effects, or include changes in sexual mixing patterns during the pandemic. It will be important to clarify the effect of the COVID‐19 pandemic on HIV self‐testing in these settings as more data becomes available.

## 5 CONCLUSIONS

The COVID‐19 emergency is impacting HIV care worldwide, as face‐to‐face consultations and laboratory testing are reduced, drug and condom manufacture and transport are interrupted, and lockdowns affect peoples’ ability to access testing or collect medicines. Gaps in HIV treatment could lead to increased deaths from HIV and further HIV transmission, placing further burdens upon healthcare systems. The overall impact of COVID‐19 on new HIV infections and HIV‐related deaths is expected to be low to moderate for MSM in China, but this is dependent on the scale and length of the various disruptions. Resources should be urgently directed to ensuring VS and condom use remains high in order to mitigate any adverse effects of COVID‐19 disruption on HIV transmission and control among MSM in China.

## COMPETING INTERESTS

KMM has received an honorarium from Gilead for speaking outside of the submitted work. All other authors have no competing interests.

## AUTHORS’ CONTRIBUTIONS

Conception and design of the study: RDB, GF, JJO, JDT, KMET, WT, PV, KMM. Acquisition of data: GF, JL, WT. Mathematical modelling: RDB, LM, KMET, PV, KMM. Coding and simulations: RDB. Analysis and interpretation of results: RDB, GF, LM, JL, JJO, JDT, KMET, WT, PV, KMM. Writing and drafting of the manuscript: RDB, GF, LM, JL, JJO, JDT, KMET, WT, PV, KMM. Approval of the submitted manuscript: RDB, GF, LM, JL, JJO, JDT, KMET, WT, PV, KMM.

## ABBREVIATONS

ART, antiretroviral therapy; CDC, centre for disease control; CI, confidence interval; COVID‐19, the disease caused by the SARS‐CoV‐2 (2019‐nCoV) coronavirus; CrI, credible interval; HIV, human immunodeficiency virus; LMIC, low‐ and middle‐income countries; MSM, men who have sex with men; PLHIV, people living with HIV; VS, viral suppression.

## Supporting information


**Table S1.** All cities combined
**Table S2.** Guangzhou
**Table S3.** Shenzhen
**Table S4.** Jinan
**Table S5.** Qingdao
**Table S6.** Key parameters and fitting metrics used in the model in Booton et al.^1^ (summary of 95% confidence interval uncertainty ranges) for Guangzhou, Shenzhen, Jinan and Qingdao cities.
**Table S7.** Uncertainty in the choice of disruption parameter A‐E (0 – 100% of value) given with the total new infections over 1 years (I1) and deaths (D1) and as percentage changes in new infections (%I1) and deaths (%D1). Upper and lower values are given for each e.g. %I1L to %I1 U gives the percentage change in new infections over 1 years. Scenarios are as follows: A) Reduction in facility‐based HIV testing, B) Reduction in ART initiation, C) Reduction in number of sexual partnerships, D) reduction in condom use, E) Reduction in viral suppression. *C100 is replaced with C95 in order to keep 5% of partnerships (the model requires some partnership information in order to function).
**Table S8.** Uncertainty in the choice of disruption parameter A‐E (0 – 100% of value) given with the total new infections over 5 years (I5) and deaths (D5) and as percentage changes in new infections (%I5) and deaths (%D5). Upper and lower values are given for each e.g. %I5L to %I5 U gives the percentage change in new infections over 5 years. Scenarios are as follows: A) Reduction in facility‐based HIV testing, B) Reduction in ART initiation, C) Reduction in number of sexual partnerships, D) reduction in condom use, E) Reduction in viral suppression. *C100 is replaced with C95 in order to keep 5% of partnerships (the model requires some partnership information in order to function).
**Figure S1.** Comparison of the model projections in Booton et al.^1^ for each city against data on HIV prevalence, incidence per 100 person years, percentage on ART if diagnosed and percentage diagnosed. Projections are shown for Guangzhou (A‐D), Shenzhen (E‐H), Jinan (I‐L) and Qingdao (M‐P), with median (black line) and 95% credible interval (blue shaded area) being displayed for 100 model fits for each city. Empty red squares indicate HIV diagnosis data which was fit to (accepted if confidence interval) and red circles indicate those data which are included in the likelihood estimation to determine the best fitting model runs. The blue dot represents validation data from 2018 (86.5% of diagnosed MSM on ART in a recently published UNAIDS report^21^).
**Figure S2.** Mathematical model schematic for Booton et al.^1^ Subscripts on uninfected (X) stages indicate diagnosis/ART stage (y); subscripts on infected (Y) stages indicate infection stage (x) and diagnosis/ART stage (y). Risk and role subscripts are omitted for clarity.
**Figure S3.** Sexual mixing with six groups by role and risk in Booton et al.^1^
**Figure S4.** Uncertainty in the choice of disruption parameter (0% to 100% of value) plotted with the two impact percentage changes in new infections and deaths over 1 years for a 3‐month disruption. Full data can be found in Table S7.
**Figure S5.** Uncertainty in the choice of disruption parameter (0% to 100% of value) plotted with the two impact percentage changes in new infections and deaths over 5 years for a 3‐month disruption. Full data can be found in Table S8.
**Figure S6.** Time‐dependent testing assumptions used in the model for facility/self‐testers and first/repeat testers for both low and high risk, with facility trend fixed to early city estimates (black). Guangzhou is shown in A‐B, Shenzhen in C‐D, Jinan in E‐F and Qingdao in G‐H. Overall trends are shown in dark blue for facility and in dark red for self‐testing.
